# Effective x-ray attenuation coefficient measurements from two full field digital mammography systems for data calibration applications

**DOI:** 10.1186/1475-925X-7-13

**Published:** 2008-03-28

**Authors:** John J Heine, Jerry A Thomas

**Affiliations:** 1 Cancer Prevention & Control Department, Moffitt Cancer Center, Tampa, Florida, USA; 2 Department of Radiology, Via Christi Regional Medical Center, Wichita Kansas, USA

## Abstract

**Background:**

Breast density is a significant breast cancer risk factor. Currently, there is no standard method for measuring this important factor. Work presented here represents an essential component of an ongoing project that seeks to determine the appropriate method for calibrating (standardizing) mammography image data to account for the x-ray image acquisition influences. Longer term goals of this project are to make accurate breast density measurements in support of risk studies.

**Methods:**

Logarithmic response calibration curves and effective x-ray attenuation coefficients were measured from two full field digital mammography (FFDM) systems with breast tissue equivalent phantom imaging and compared. Normalization methods were studied to assess the possibility of reducing the amount of calibration data collection. The percent glandular calibration map functional form was investigated. Spatial variations in the calibration data were used to assess the uncertainty in the calibration application by applying error propagation analyses.

**Results:**

Logarithmic response curves are well approximated as linear. Measured effective x-ray attenuation coefficients are characteristic quantities independent of the imaging system and are in agreement with those predicted numerically. Calibration data collection can be reduced by applying a simple normalization technique. The calibration map is well approximated as linear. Intrasystem calibration variation was on the order of four percent, which was approximately half of the intersystem variation.

**Conclusion:**

FFDM systems provide a quantitative output, and the calibration quantities presented here may be used for data acquired on similar FFDM systems.

## 1. Background

Early detection is a key element in reducing breast cancer mortality [1]. Mammography screening is an essential surveillance component for early detection [2]. Similarly, there is interest in developing total cancer care methods in clinical practice so that disease screening and treatment can be tailored to the patient [3]. The development of accurate breast cancer risk models may play an important role in designing risk based cancer control strategies. Because breast density is a significant breast cancer risk factor [4], it may be useful to include it in the clinical setting for risk assessment. The Gail breast cancer risk model is used for intervention studies and counseling [5] but does not include breast density beyond research purposes. There is a critical need to incorporate all available information on breast cancer risk to ensure that risk models are useful for clinical decision making [6].

Mammographic density and breast density are terms used synonymously to describe the degree of bright areas in mammograms, which is related to dense breast tissue. Substantial evidence indicates that women with greater quantities of breast density have a significantly increased breast cancer risk [7]. Breast density may be assessed from either film mammograms or from the newer full field digital mammography (FFDM) systems. To date, most published breast density related research is based on film analysis without applying standardization [4,8], which may be due to the availability of archived film data relative to FFDM data. The overall equivalence of film mammography and FFDM for breast cancer detection [9] may indicate that the FFDM system use will increase, which is the case at this center.

Methods used for assessing breast density may be loosely grouped into (1) techniques that do not consider the acquisition influences, and (2) radiometric standardization techniques that compensate for the acquisition influences. Standardization (calibration) methods are to correct for interpatient variations in the x-ray exposure, beam type, compression height, and the detector response. Standardization techniques are under development for both film [10-12] and FFDM applications [13]. To the best of our knowledge, there are no published reports on calibrated techniques with risk assessments using breast cancer as the endpoint. A non-calibration user-assisted approach has been shown consistently to produce a measure that correlates with breast cancer [4,8], which produces a binary labeled image defining dense and fatty tissue. However, there is no universal standard used for measuring breast density [14].

Our previous work showed that it is possible to make effective attenuation coefficient measurements using the General Electric (GE) FFDM system with phantom imaging [8]. In this prior work, theoretical arguments were developed without considering the detector interaction or specific x-ray spectral form, which predicted linear logarithmic response curves. Idealized x-ray equations were then used with simulated x-ray spectra, tabulated attenuation data, and numerical integration to generate logarithmic response curves. These numerically generated curves were well approximated as linear and were used to make effective attenuation coefficient estimates, which were then compared with measured coefficient values obtained experimentally with phantom imaging. This work suggested that the effective attenuation coefficients may be used for calibration purposes as known quantities. This previous work did not provide measured effective attenuation coefficient values for glandular or fatty breast tissue.

Findings presented here represent essential components of an ongoing project for developing calibration techniques for FFDM breast cancer risk applications [8] that extends earlier research [13]. This project includes measuring the intra/inter system concordance of calibration data generated with phantom imaging using two similar FFDM systems. Serial stability comparisons will be analyzed after collecting sufficient data over an extended period. The overall goals of this multiyear project are to (1) determine the necessary amount of calibration data that must be collected (sampled) to calibrate the prospective data with a given accuracy using breast cancer as the endpoint, (2) analyze the serial stability of the systems by sampling the calibration data, and (3) study the wider applicability of calibration data generated on a given system by assessing the necessary requirements to merge data from multiple systems. The serial stability analysis is in support of a multiyear case control study that involves collecting prospective patient image/risk data. By hypothesis, this data will be used to validate the calibration procedure by showing that it produces a stronger risk relation when compared with methods that do not consider the acquisition influences when applied to the same dataset.

Specifically in this report, effective attenuation coefficients and breast tissue equivalent calibration curves generated with two similar FFDM systems were compared for the three filter\target combinations and multiple voltage settings. This may be considered the baseline (BL) data collection and analysis for the ongoing serial study. Effective attenuation coefficients were also compared with values generated from numerical integration by using idealized x-ray attenuation equations. Methods for generating these quantities were described previously [8] and will not be repeated in detail here. A technique for reducing the amount of calibration data collection was investigated.

The percent glandular (*PG*) equivalent transform (map) construction and functional form were investigated with a specific acquisition example and numerical methods. In practice, the *PG *map will be used to calibrate arbitrary image data. Tissue equivalent logarithmic response curve analyses enabled the map construction. Uncertainty in the *PG *map was estimated with error propagation techniques.

## 2. Methods

**Figure 1:**
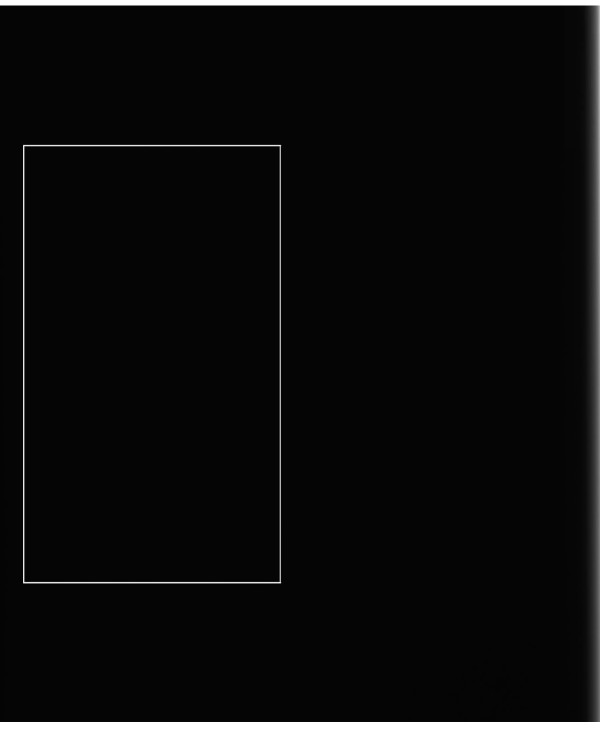
**Rectangular phantom used in the Florida location.** Labeled rectangular region is the field of view defined in the manuscript. The phantom nearly covers the entire detector.

**Figure 2:**
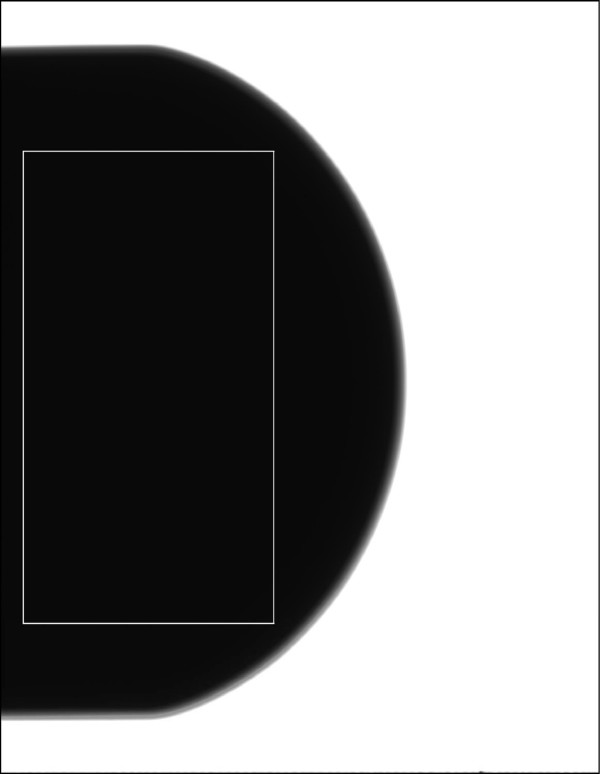
**Semicircular phantom used in the Kansas location.** The interior rectangle covers the same detector region as the field of view defined in Fig 1. Because the off phantom area is over exposed, the outside detector perimeter was labeled manually for reference.

Phantom imaging was performed at two locations with GE Senographe 2000D FFDM systems that were manufactured about the same time (spring-summer 2000). Both systems are used for clinical breast screening. The systems are located at the Moffitt Cancer Center, Tampa, Florida, and the via Christy Regional Medical Center, Wichita, Kansas, which will be referenced as the FL and KS locations, respectively. Senographe 2000D systems produces both raw data (x-ray exposure representation) and processed data with 100 micron digital resolution and 14 bit dynamic pixel range. Raw data was used for this work. This system has three target\filter combinations (described below). The system detector is described elsewhere [13,15]. Phantoms used for this study were obtained from Computerized Imaging References Systems (CIRS, Norfolk VA). The quality of these phantoms and their close similarity with the x-ray interaction characteristics of breast tissue within the realm of mammography x-ray spectra are discussed in previous work [8] and related references therein. Phantoms for both locations have different geometries but were manufactured with the same material specifications: 100% glandular breast tissue equivalent and 100% fatty breast tissue equivalent. Florida phantoms are 18 cm × 24 cm rectangular slabs with either one or two cm heights. Kansas phantoms have a semicircular breast-like geometry with one cm heights. Example phantom images for the FL and KS locations are shown in Fig. 1 and Fig. 2, respectively. Kansas phantoms are 19 cm in length along the left hand margin in the *y *direction (from top to bottom), and the distance from left hand border along the *x *direction to the furthest distance to the semicircular perimeter is 13 cm.

Baseline tissue equivalent logarithmic response curves were measured for both locations. Response curves were generated by measuring the response for a particular acquisition technique (for a given voltage setting, target\filter combination, and exposure) as a function of phantom configuration height above the detector. These response curves permit the calibration of data collected with other acquisition techniques. Molybdenum/Molybdenum (Mo/Mo), Molybdenum/Rhodium (Mo/Rh), and Rhodium/Rhodium (Rh/Rh) target\filter combinations were used to generate the response curves. The voltage setting abbreviation used here corresponds with the peak voltage (kVp) system console indicator. Each tissue type response curve was generated for these settings: Mo/Mo – [26,27] kVp; Mo/Rh – [28, 29] kVp; and Rh/Rh – [30 kVp]. All BL response curves were generated with the 160 milliamp second beam-current system setting, which corresponds to the system console *mAs *indicator. The system *mAs *value was used as surrogate for the incident exposure. Phantoms were imaged either with [2–6] cm, [2–7] cm, or with [3–7] cm heights depending on the target\filter combination using one cm height increments. The 160 *mAs *setting was used because it provided signal when imaging the more attenuating [6–7] cm glandular configuration while not saturating the intensity values for less attenuating [2–3] cm fatty phantom arrangements. All image data was acquired as left cranial caudal views. Florida BL data was acquired in October 2006, and KS data was acquired in November 2006. The compression paddle was in place and in contact with the top phantom for all configurations while imaging to mimic patient imaging. The flat field quality assurance procedure (assessed by the built-in system diagnostics) was implemented before imaging to ensure that the system was operating within the manufacturer's tolerances.

An 800 × 1400 pixel rectangular region was used for the analysis, which defined the field of view (FOV). The aim was to restrict the comparison analysis to the same detector regions for both systems. The FOV, which is shown in Fig. 1 and Fig. 2, was dictated by the KS phantom geometry. The FOV is approximately the largest rectangle that fits within the semicircular geometry with the left hand margin.

Logarithmic response (*LR*) curves were determined for each 50 × 50 pixel sector within the FOV as a function of phantom height and averaged for a given tissue type. Each sector was fitted with this linear model

*LR*(*t*) = -*μt *+ *l*,

where *t *is a given phantom configuration height measured in cm, μ is the effective x-ray attenuation coefficient for a given tissue type measured in cm^-1^, and *l *is the logarithmic intercept value (unitless) for a given tissue type. Within a given sector, the pixel values (*V*) were averaged and natural logarithm transformed. This procedure was applied to each phantom for given height, given sector, and then for every sector and height. Plotting ln [mean(*V*)/*mAs *], where *mAs *is the system acquisition readout, as a function of phantom height above the breast support surface (bucky) gives the empirical *LR *for a given square. The *mAs *normalization is discussed below in more detail. The sectored analysis resulted in a distribution of 16 × 28 values for each of the two model parameters for each tissue type *LR *curve. Findings will be presented as the mean and standard deviation for the various distributions. Confidence intervals (CIs) were generated for the regression parameters. For one example, the *LR *plot will be provided for one individual sector centered on the detector in the *y *direction positioned on the left-hand side of FOV (approximately under the focal point). The R^2 ^statistic was used to assess the linear model agreement, because it is the fraction of variability explained by the model (unity implies perfect agreement) and therefore represents an important goodness of fit measure derived from the residual analysis. The distribution of R^2 ^coefficients was generated and summarized to assess the linear model agreement. Effective attenuation coefficients were derived with numerical methods discussed previously [8] and compared with the corresponding measured values.

Previously [8] for normalization purposes, the detector response was first measured as function of beam-current setting and fitted to this model: *V *= *m*(*mAs*) + *b*, where *m *and *b *are constants for a given beam-type (fixed kVp and target\filter settings) [8]. This was achieved by acquiring exposures of the detector (no attenuation) for *mAs *settings up to the point of detector saturation (not including saturation). Response curves acquired with phantom imaging were analyzed by first transforming *V *to the equivalent *mAs *value for a arbitrary acquisition by inverting the detector response: *mAs *equivalent = (*V*-*b*) *m*^-1^. The equivalent *mAs *value was then normalized by the acquisition *mAs *and logarithmically transformed giving the logarithmic relative exposure relation: *LRE *= ln[(*V *- *b*) (*mAs *× *m*)^-1^]. The response generated with the reference *mAs *was then equated with the response generated for arbitrary *mAs *value giving

$$\ln[\frac{V_{r}-b}{mAs_{r}\times m}]=\ln[\frac{V-b}{mAs\times m}],$$

where the subscript indicates reference values. When *V *> *b*, this relation reduces to

$$\ln[\frac{V_{r}}{mAs_{r}}]\approx \ln[\frac{V}{mAs}].$$

For the work below, this normalization was applied

$$LR=\ln[\frac{V}{mAs\times m}]\sim \ln[\frac{V}{mAs}],$$

where *V *is the average sector value. When the Eq. (3) approximation holds, the need to characterize the detector response is eliminated. Moreover, Eq. (3) suggests that for fixed voltage setting and target\filter combination, a response curve generated with one *mAs *value is equivalent to all other curves acquired with varying *mAs *values. In this work, Eq. (4) was measured as a function of phantom height and fitted with the linear model defined in Eq. (1) for both tissue types.

For the *mAs *normalization analysis, additional phantom imaging was performed at the FL location (only) to generate the Mo/Mo – 26 kVp glandular tissue *LR *curves for 160 *mAs *and 200 *mAs *acquisitions to assess Eq. (3). Additional data was also acquired to generate the fatty tissue *LR *curves for 160 *mAs *and 110 *mAs *for the same voltage setting and target\filter. In this analysis, *mAs*_r _= 160. This additional data collection was used to eliminate the possibility of serial drift influence in the normalization analysis. Mo/Mo – 26 kVp acquisition parameters are approximately the average settings used in clinical practice [16].

To assess the linear calibration model, the calibration procedure derived from the *LR *regression parameters was compared with the measured calibration curve for a fixed height of 6 cm (*T *= 6 cm total height). For a given acquisition technique, two calibration points are required [13] to calibrate arbitrary image data. These points correspond to the *LR *value for each tissue type for a given height, which can be estimated from Eq. (1) with *t *= *T*. In practice, the detected *LR *value is likely to fall between the endpoints. For fixed height and arbitrary logarithmic response, *LR*_a _= *z*, the *PG *map is given by

*PG *= *Mz *+ *B*.

The validity of the linear map form is addressed in the Appendix. The *M *and *B *parameters can be expressed as functions of the *LR *regression parameters by using *PG *= 100% and *PG *= 0% as boundary conditions with Eq. (1), which gives

*M *= 100 × [(*μ*_*g *_- *μ*_*f*_)*T *+ (*l*_*g *_- *l*_*f*_)]^-1^

and

$$B=\frac{1}{2}(100-M\times [(\mu_{g}+\mu_{f})T+(l_{g}+l_{f})]),$$

where the *g *and *f *subscripts denote glandular and fatty tissue values, respectively. Equation 1 was modified to derive Eq. (6) with this form: *LR *= *μt *+ *l*. A sequence of five phantom images was acquired to verify Eq. (6). This sequence represents a surrogate composite fatty/glandular tissue mixture for *T *= 6 cm corresponding to the known [0, 16.6, 50, 83.3, 100] *PG *mixtures. The known *PG *is determined from this relation: (height of the 100% glandular tissue component)/*T *× 100% for a given phantom image in the sequence. In [glandular, fatty] cm component phantom heights, these proportions correspond to composite heights of [0,6], [1,5], [3,3], [5,1], and [6,0], where the two component heights within a given bracket add to 6 cm. The sequence represents a surrogate because the various mixtures are not present in one image but in five separate images acquired consecutively. As a specific example, the Mo/Mo – 26 kVp acquisition settings were used to generate the sequence. Plotting *z *(see Eq. (5)) along the *x *axis and plotting the known *PG *values along the *y *axis shows the map. The agreement between *PG *= *Mz *+ *B *using the *LR *regression distribution quantities substituted into Eq. (6) was compared with the known and measured *PG *determined from the surrogate composite phantom images.

For the map analysis, *LR*s were generated again for [2–6] cm heights using the same phantom imaging procedure outlined above by acquiring another dataset not included in the BL acquisition. This additional data collection will show the best scenario because the data (the phantom images for the tissue *LR*s and *PG *curves) were collected on the same day in January 2007 to eliminate the possibility of serial drift uncertainty. A sector example will also (the same sector as above) be used for demonstration purposes.

Variation in the map due to the uncertainty in each quantity may be estimated with derivative approximations using *PG *= *f*(μ_g_, μ_f_, *l*_g_, *l*_f_, z) giving

$$\begin{array}{l} {\left(\Delta PG\right)}^{2}=|\frac{\partial f}{\partial \mu_{g}}\Delta \mu_{g}{|}^{2}+|\frac{\partial f}{\partial \mu_{f}}\Delta \mu_{f}{|}^{2}+\\ |\frac{\partial f}{\partial l_{g}}\Delta l_{g}{|}^{2}+|\frac{\partial f}{\partial l_{f}}\Delta \mu_{f}{|}^{2}+|\frac{\partial f}{\partial z}\Delta z{|}^{2}, \end{array}$$

where the Δ quantities are the estimated variation in the respective parameters. The explicit terms for Eq. (8) are derived in the Appendix using Eq. (6) with Eq. (7). The height, *T*, is considered as exact and therefore does not contribute. The quadratic form used in Eq. (7) assumes each component contribution is independent. Equation (7) was used to estimate both intersystem variability (external variation) and intrasystem variability (internal variation). The total variation is given by

$$\Delta PG={\left[n_{1}^{2}+n_{2}^{2}+n_{3}^{2}+n_{4}^{2}+n_{5}^{2}\right]}^{1/2},$$

where each term in Eq. (7) has been relabeled, respectively. Intersystem analysis was used to derive the Δ terms for the external variation, which provided an estimate of the uncertainty when applying the map generated at one facility for data collected at another location. The internal variation was estimated with the FL spatial *LR *analysis. For both internal and external analyses, z and *Δz *were estimated for each *PG *surrogate component (6 cm height) as the average and standard deviation of log-transformed and *mAs *normalized FOV pixel distribution, respectively. Although *z *and Δz were estimated with FL data, they support reasonable estimates of the anticipated uncertainty due to the variations in the other *PG *map parameters for both uncertainty forms. The 26 kVp – Mo/Mo acquisition was used for the uncertainty analysis.

## 3. Results

### 3. 1 Inter and intra system comparisons

Table 1 shows the regression parameter distribution summary quantities for Mo/Mo datasets. The 95% two sided CIs for the effective attenuation coefficients and intercepts are provided below the respective quantities for these and the other examples. The CIs represent averages taken over all sectors for the distribution examples. For a given tissue type, effective x-ray attenuation coefficients are similar between locations, whereas *l *values vary somewhat relative to attenuation coefficient variation. Agreement may be gauged from the parenthetical entries and CIs. Parenthetical quantities listed in the attenuation coefficient and intercept columns are absolute values of the percent difference between the location parameters. For example, the glandular quantity was calculated as |(μ_gFL _- μ_gKS_)/μ_gKS_| × 100%, which was used for all calculations of this type. For either location, the intrasystem variation is relatively small for the regression parameters, which follows from the σ and σ_l _column entries. Glandular tissue attenuation coefficient variation is larger than the corresponding fatty tissue variation as indicated in the Δ column. Signal is attenuated greater at a given height for the glandular tissue relative to the fatty tissue indicating there may be greater uncertainty due to the decreased signal. The R^2 ^statistic for all cases is close to unity indicating agreement with the linear model.

**Table 1:** Molybdenum/Molybdenum regression distribution quantities. Quantities are summaries of the field of view: μ is the mean effective x-ray attenuation coefficient, σ is the standard deviation of the μ distribution, Δ is the mean variation in μ derived from averaging the individual regression variation, *l *is the mean logarithmic intercept, σ_*l *_is the standard deviation of the *l *distribution, and R^2 ^is the mean of the associated distribution. Two sided 95% mean confidence interval is cited under the respective μ and *l *quantities. Location is indicated by the subscripts of the left hand column entries. Parenthetical FL entries indicate the absolute value percent difference between respective location quantities.

Mo/Mo 26 kVp	μ	σ	Δ	*l*	σ_l_	R^2^
Fat_FL_	0.572	0.001	0.005	5.093	0.010	0.998
	0.557–0.586 (1.9%)			5.03–5.15 (2.6%)		
Fat_KS_	0.583	0.001	0.005	4.964	0.010	0.998
	0.568–0.599			4.89–5.03		
Gland_FL_	0.833	0.002	0.015	4.893	0.001	0.998
	0.784–0.882 (0.60%)			4.68–5.10 (2.6%)		
Gland_KS_	0.838	0.010	0.012	4.767	0.020	0.998
	0.799–0.877			4.60–4.93		

Mo/Mo 27 kVp	μ	σ	Δ	*l*	σ_l_	R^2^

Fat_FL_	0.559	0.001	0.005	5.262	0.010	0.999
	0.544–0.575 (2.1%)			5.19–5.32 (1.8%)		
Fat_KS_	0.571	0.001	0.005	5.165	0.010	0.999
	0.554–0.586			5.09–5.16		
Gland_FL_	0.805	0.003	0.020	5.043	0.010	0.998
	0.750–0.860 (0.40%)			4.81–5.28 (1.9%)		
Gland_KS_	0.808	0.005	0.014	4.950	0.020	0.999
	0.763–0.854			4.75–5.14		

**Figure 3:**
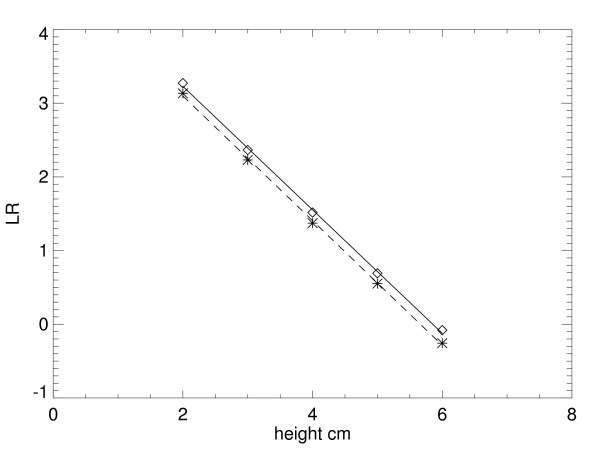
**Glandular tissue logarithmic response sector example plots.** These plots show the example sector response curves for the Florida (diamonds) and Kansas (asterisks) locations for the 26 kVp – Mo/Mo settings. The fitted regression line is solid for Florida and dashed for Kansas.

**Figure 4:**
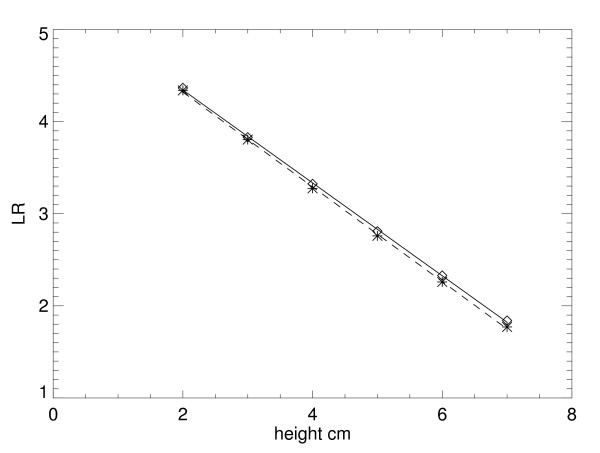
Fatty tissue logarithmic response sector example plots (same format as Fig. 3).

Variation in R^2 ^is not provided because it was very close to zero in all cases. Individual sector *LR *example plots for the Mo/Mo – 26 kVp settings are shown in Fig. 3 (glandular) and Fig. 4 (fatty). Plots for a given tissue type are shown in the same figure for intersystem comparisons. In these plots, measured points from the FL location are denoted by diamonds and asterisks for the KS measured points. Fitted lines from the regression analysis are solid for the FL plots and dashed for KS plots. Regression parameters are listed in Table 2. Regression parameter distribution quantities for the other Mo/Rh and Rh/Rh settings are shown in Table 3. The corresponding individual sector plot examples for these other settings are not shown because they were similar to the prior examples. For all distribution examples, variation within given location is relatively small, and the effective attenuation coefficients are in close agreement between the locations. Moreover, the linear model approximation holds when assessing the R^2 ^statistic for all situations. To show the intersystem agreement, a paired t-test was applied by pairing all of the 10 average attenuation coefficient quantities for the FL location with the corresponding values from the KS location (second column from Table 1 and Table 3), which gave *p *= 0.56 (linear correlation of = 0.97). Following the same procedure for the 10 average intercept quantities (using the fifth columns from the same tables), gave *p *= 0.101 (linear correlation = 0.99). Using the *p *value as a gauge, effective attenuation coefficients as an ensemble are similar across the two systems. Intersystem intercept comparisons indicate a weak system dependence.

**Table 2:** Molybdenum/Molybdenum individual sector example regression parameters. Labeling is analogous to Table 1 as applied to a single realization.

Mo/Mo 26 kVp	μ	Δ	*l*	R
Fat_FL_	0.573	0.004	5.103	0.999
	0.559–0.586 (2.4%)		5.05–5.16 (2.4%)	
Fat_KS_	0.587	0.005	4.99	0.999
	0.573–0.600		4.92–5.04	
Gland_FL_	0.837	0.014	4.904	0.999
	0.791–0.883 (0.12%)		4.70–5.09 (2.4%)	
Gland_KS_	0.845	0.011	4.788	0.999
	0.809–0.882		4.59–4.98	

**Table 3:** Molybdenum/Rhodium and Rhodium/Rhodium regression distribution quantities (using the same format as in Table 1).

Mo/Rh 28 kVp	μ	σ	Δ	l	σ_l_	R^2^
Fat_FL_	0.503	0.002	0.005	5.343	0.011	0.999
	0.490–0.516 (1.9%)			5.28–5.40 (0.1%)		
Fat_KA_	0.511	0.001	0.005	5.34	0.010	0.999
	0.498–0.525			5.27–5.40		
Gland_FL_	0.718	0.002	0.013	5.155	0.013	0.998
	0.683–0.753 (1.0%)			4.98–5.32 (1.0%)		
Gland_KS_	0.711	0.010	0.012	5.118	0.020	0.999
	0.677–0.745			4.95–5.28		
Mo/Rh 29 kVp						
Fat_FL_	0.495	0.002	0.004	5.47	0.011	0.999
	0.482–0.507 (1.2%)			5.41–5.53 (0.20%)		
Fat_KA_	0.501	0.001	0.004	5.48	0.010	0.999
	0.489–0.513			5.42–5.54		
Gland_FL_	0.700	0.002	0.014	5.274	0.014	0.998
	0.661–0.739 (1.2%)			5.08–5.46 (0.20%)		
Gland_KS_	0.692	0.010	0.013	5.263	0.020	0.998
	0.655–0.729			5.08–5.44		
Rh/Rh 30 kVp						
Fat_FL_	0.442	0.001	0.002	5.648	0.010	0.999
	0.435–0.449 (0.21%)			5.61–5.68 (1.1%)		
Fat_KA_	0.443	0.001	0.002	5.712	0.010	0.999
	0.434–0.451			5.66–5.75		
Gland_FL_	0.644	0.002	0.010	5.557	0.010	0.999
	0.621–0.666 (2.4%)			5.45–5.66 (1.0%)		
Gland_KS_	0.629	0.005	0.010	5.593	0.014	0.999
	0.609–0.648			5.49–5.69		

Measured attenuation coefficients were compared with those generated with numerical methods. The resulting figures of merit are listed in Table 4. The last column gives the averaged intersystem measured attenuation coefficients for comparison purposes. The R^2 ^values are also provided for this modeling, which indicate the linear model approximation holds in the theoretical developments as well. Numerical effective attenuation values are listed in the μ_*T *_column. The associated CIs indicate approximate agreement with the measured values. Measured value agreement is also indicated by the parenthetical percent differences cited with the respective *μ*_T _values. Intercept quantities were irrelevant.

**Table 4:** Effective attenuation coefficient comparisons. This shows the numerically derived attenuation coefficients, μ_T_, the associated regression variation, Δ, and R^2 ^values for the Mo/Mo, Mo/Rh, and Rh/Rh configurations for the same voltage settings (kVp) used for the phantom imaging. Attenuation coefficient confidence intervals are cited beneath the respective values. The <*μ *> column gives the averaged FL + KS measured attenuation coefficients for the respective tissue types. Percent differences between < μ > and *μ*_T _are listed parenthetically next to the respective *μ*_T _values. Other formatting is the same used above.

Mo/Mo	*μ*_T_	Δ	R^2^	<*μ *>
Fat_26_	0.595 (2.8)	0.010	0.999	0.578
	0.575–0.613			
Gland_26_	0.871 (4.1)	0.020	0.998	0.835
	0.818–0.923			
				
Fat_27_	0.585 (2.9)	0.010	0.999	0.565
	0.562–0.604			
Gland_27_	0.836 (3.4)	0.021	0.997	0.807
	0.777–0.894			
Mo/Rh				
Fat_28_	0.529 (4.0)	0.010	0.999	0.508
	0.511–0.547			
Gland_28_	0.764 (6.8)	0.014	0.998	0.713
	0.772–0.807			
Fat_29_	0.516 (3.8)	0.010	0.999	0.498
	0.498–0.535			
Gland_29_	0.735 (5.3)	0.020	0.998	0.696
	0.690–0.779			
Rh/Rh				
Fat_30_	0.462 (4.3)	0.010	0.999	0.442
	0.448–0.476			
Gland_30_	0.675 (5.7)	0.010	0.999	0.636
	0.647–0.703			

### 3. 2 Response normalization

Table 5 shows the additional *LR *distribution parameters generated for the *mAs *response normalization analysis using [160, 110] *mAs *for the fatty tissue type and [160, 200] *mAs *for the glandular tissue type. For each tissue type, *mAs*_r _= 160. The quantities in Table 5 show that the Eq. (3) approximation holds. A comparison of the respective attenuation coefficients for the varying *mAs *values indicates close agreement. This suggests the *LRs *acquired for a given acquisition *mAs *determine the *LR*s for an arbitrary *mAs *readout value.

**Table 5:** Normalization regression parameters. This gives the logarithmic response curve fitted parameter Molybdenum/Molybdenum (Mo/Mo) distribution quantities for both tissue types using the beam-current readout normalization (*mAs*). Acquisition *mAs *system values are indicated by the subscript of the left column entries.

Mo/Mo 26 kVp	μ	σ	Δ	l	σ_l_	R^2^
Fat_160_	0.568	0.002	0.005	5.054	0.01	0.999
	0.550–0.586			4.97–5.13		
Fat_110_	0.569	0.002	0.006	5.058	0.010	0.999
	0.549–0.590			4.97–5.14		
Gland_160_	0.827	0.002	0.016	4.851	0.010	0.998
	0.777–0.878			4.63–5.07		
Gland_200_	0.826	0.002	0.015	4.853	0.010	0.998
	0.777–0.875			4.64–5.06		

### 3.3 Percent glandular map

**Figure 5:**
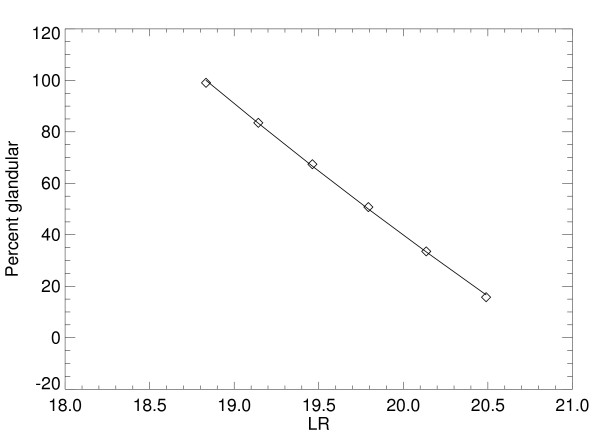
**Numerically derived percent glandular map.** This corresponds to the example shown in The regression line is solid and numerically generated points are diamonds (see Appendix). In this case R^2 ^= 0.999, which shows the map is well approximated as linear.

**Figure 6:**
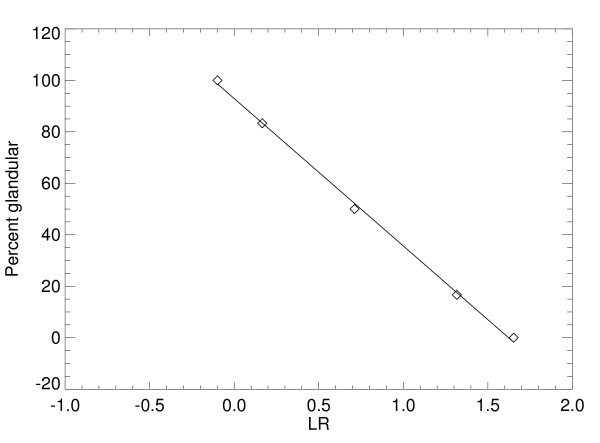
**Percent glandular map individual sector example for the Mo/Mo – 26 kVp settings.** The (logarithmic response = *z*, theoretical percent glandular composition) ordered pairs are represented by diamonds, and the (*z*, regression fitted value) ordered pairs are represented by a solid line. Absolute difference between the known and fitted values was 1.7% for all points. This corresponds with the map shown in Fig. 5.

Calibration map distribution quantities derived from the surrogate mixture phantom images are listed in the *PG *row of Table 6. Internal variation in *M *is small relative to the best estimate of *M*, spatial variations in both *M *and *B *are relatively small, and R^2 ^indicates the linear model approximation holds. The bottom row of Table 6 shows *M *and *B *estimated with Eq. (6) using the quantities from Table 5. Deviation of the measured *PG *value from the known phantom configuration is discussed below. Quantities in Table 6 indicate that the measured map agrees with the model and is well approximated as linear, as predicted by the work in the Appendix; a related numerically generated map is shown in Fig. 5. As further demonstration, the measured map determined from the individual sector example is shown in Fig. 6. For this case, *M *= -57.23 ± 1.23, *B *= 93.93 with R^2 ^= 0.999. Both *M *and *B *values for the sector agree with the respective entries in Table 6.

**Table 6:** Percent glandular map parameters. The percent glandular row shows distribution quantities for the Molybdenum/Molybdenum (Mo/Mo) map determined with surrogate mixture phantom imaging. The PG_160 _row gives *M *and *B *calculated with Eq. (7) using the 160 *mAs *regression parameters from Table 5 with Eq. (6).

Mo/Mo 26 kVp	M	σ_M_	Δ	B	σ_B_	R^2^
PG	-57.86	0.31	1.15	94.45	0.77	0.998
	-61.5 – -54.2			90.7–98.2		
PG_160_	-57.07	/	/	93.76	/	/

### 3.4 Uncertainty analysis

Uncertainty in the *PG *components expressed in Eqs. (7–8) were generated for the intersystem variation (external) and the intrasystem (internal) variation for a Mo/Mo – 26 kVp example. External variations in the log intercepts and attenuation values were estimated by taking the absolute value difference between respective location quantities from Table 1. The 160 *mAs *generated effective attenuation coefficients and the log intercepts listed in Table 5 were used as the known values for both the external and internal variation calculations. Internal variation was estimated by using the 160 *mAs *spatial distribution values cited in Table 5. Internal variations in the attenuation coefficients and log intercepts were taken from the σ and σ_l _columns in Table 5. Estimated uncertainties for the regression analysis parameters are summarized in Table 7. Uncertainties are broken down into their respective components along with the totals in Table 8, which follow from Eqs. (7–8); *z *distribution quantities are cited in the bottom rows. External variation is approximately twice that of the internal. External logarithmic intercept differences represent major contributions, which are cited in the n_3 _and n_4 _columns; as one component increases the other decreases and vice versa, which maintains the variation throughout the *PG *composition. Related internal components show a similar trend but with smaller contributions. If a bias exists with this specific example, it will tend to over estimate the external variation because this dataset has at least equivalent if not more intersystem variability in comparison with the other datasets.

**Table 7:** Estimated uncertainties for the Molybdenum/Molybdenum example.

Mo/Mo 26 kVp	Δμ_g_	Δ*l*_g_	Δμ_f_	Δ*l*_f_	ΔLR
external	0.01	0.130	0.01	0.140	0.15
internal	0.002	0.01	.002	0.01	0.012

**Table 8:** Percent glandular map absolute % variation. This shows the individual external error (top) and internal error (bottom) components for the Molybdenum/Molybdenum – 26 kVp map output for each mixture. Totals follow from Eq. (8). The last two rows list the field of view average and standard deviation for each composition.

PG external variation	0	16.6	50	83.3	100
n_1_	0.02	0.30	0.89	1.42	1.68
n_2_	3.8	3.1	1.8	0.64	0.07
n_3_	0.07	1.3	3.7	6.0	7.0
n_4_	7.4	6.0	3.5	1.3	0.14
n_5_	0.78	0.95	1.42	1.9	2.3
total external variation	8.4	7.0	5.7	6.6	7.6
Internal variation					
n_1_	0.02	0.03	0.89	1.42	1.68
n_2_	3.80	3.10	1.81	0.64	0.08
n_3_	0.0	0.10	0.30	0.47	0.56
n_4_	0.58	0.47	0.30	0.10	0.01
n_5_	0.78	0.95	1.42	1.90	2.3
total internal variation	3.9	3.3	2.5	2.5	2.9
<z>	1.659	1.329	0.735	0.188	-0.738
Δz	0.013	0.016	0.024	0.033	0.040

## 4. Discussion

Calibration curves are well approximated as linear, which differs from related work that used polynomial modeling [13]. Differences may stem from the elemental sector size used to generate the response curves. The related work did not sector the detector. Intersystem effective attenuation coefficients are similar and are in agreement with the numerically/theoretically derived values indicating that the Senographe 2000D produces a quantitative output. Although subject to further verification, intersystem agreement is an indication that attenuation coefficients are characteristic quantities that may be saved or used as known quantities. On the other hand, intercept values are weak functions of the FFDM system. Assuming that the x-ray tube outputs are similar, differences in the intercepts may be due to variation within the detector/exposure response. To explore this hypothesis, the open detector response curves were generated for each system for the 26 kVp – Mo/Mo settings with methods described previously [8] and briefly in the Methods Section (see normalization discussion). Table 9 shows the slope and intercepts distribution values for both systems (sectored analysis). The *V *response for the KS system is about 1.6 (ratio of the slopes) that of the FL system. The KS detector saturates near 60 *mAs*, whereas the FL system saturates slightly below 100 *mAs *for this example. Intercept confidence intervals both span zero but the KS width is much wider than the FL width (about 114 in comparison with 451).

**Table 9:** Detector response parameter comparison. This shows the detector response regression parameters (distribution quantities) for both detectors for the Molybdenum/Molybdenum – 26 kVp acquisition settings fitted to *V *= *m*(*mAs*)+*b*. Parenthetical entries are distribution standard deviations.

	Slope (*m*)	Location	Intercept (*b*)	Intercept CI	R^2^
FL	183.2 (1.22)	180.9–185.4	58.8 (12.1)	-55.3–173.1	0.999
KS	300.0 (3.36)	286.5–313.5	256.1 (14.2)	-195.6–707.8	0.999

The work shows that there is relatively little spatial variation in the two regression parameters for a given location. The choice of sector size will require further analysis to determine the optimal size because it influences the variation. Without a clinical endpoint comparison (cancer/no cancer patients), the appropriate spatial resolution of the mapping cannot be determined. The optimal resolution will be determined by its ability to assign breast cancer risk after accruing sufficient patient data. Likewise, the normalization work further demonstrates that when the voltage setting and target/filter are held constant, only two calibration curves are necessary, which permits a considerable reduction of *mAs *calibration data collection (sampling). Numerical evidence developed in the Appendix shows that the map is well approximated as linear, which is supported by experimental *PG *analysis. Moreover, the calibration map generated by the calibration curve parameters is in close agreement with the measured values obtained from the surrogate composite phantom imaging, which is another indication that these FFDM systems provide a quantitative output. As the *PG *external variation analysis showed, intercepts are source of uncertainty in the map. A similar argument would apply to a given system if the system drifts in time relative to the calibration curves. If the regression parameters are stable over time and the intercept variation proves acceptable, the work suggests that calibration parameters determined on a given GE-FFDM system will apply to other similar systems without the need for additional phantom imaging if the variation estimated here is acceptable. To the best of our knowledge, the calibration values presented here are not yet available in the public domain. This analysis was limited to two like systems due to availability. However, the calibration quantities presented here may serve as reference for comparisons of related work in the field implemented on other FFDM systems or can be used in calibration research performed at other sites without additional imaging. These findings extend the previous work [8,13] by demonstrating the relationship between the calibration map, the measured calibration points, and the associated uncertainty.

## 5. Conclusion

Data used for this study is insufficient to assess the natural serial variation in the regression parameters. Longer term goals are to compare the inter/intra serial uncertainty with this BL data. Control measures from the BL data have been sampled on a biweekly basis since October 2007. Future work will use this serial data to determine the serial variation and its impact on the calibration procedure when assigning breast cancer risk to patient data. If the calibration approach is serially stable in the forward direction, it implies that data collected previously can be calibrated. It is often thought that FFDM data does not suffer from the same technical difficulties associated with film data due to varying response curves for example. This assumption may be generally true but will require validation for quantitative calibration type measurements.

Ideally, generating calibration curves for all possible acquisition techniques should produce the most accurate data normalization, which is obviously not practical. Thus, some form of interpolation is necessary. In part, the longer term aim of this work is to minimize the amount of a priori collected calibration data while maintaining accurate standardization. Showing that the effective attenuation coefficients are characteristic quantities and demonstrating the *mAs *normalization represent incremental steps in this direction. The non-specialty center calibration application acceptance (or usage) may be enhanced if the calibration data collection is minimized. In analogous fashion, future work includes minimizing the number of sample points in the voltage setting space. The guiding principle is that if the calibration requires minimal experimental effort, it may gain wider usage in the future.

## Competing interests

The author(s) declare that they have no competing interests.

## Authors' contributions

JJH performed the imaging experiments at the Florida site, developed the calibration/analysis software, performed the theoretical/numerical work, and is the primary author. JAT performed the imaging experiments at the Kansas site, developed the imaging protocol, and is the second author. Both authors have read the manuscript.

## Appendix

### A. 1 Error propagation

Explicit calculation for Eq. (8) is derived using *f *= *M*(*μ*_*g*_, *μ*_*f*_, *l*_*g*_, *l*_*f*_)*z *+ *B*(*μ*_*g*_, *μ*_*f*_, *l*_*g*_, *l*_*f*_), where *z *is an arbitrary *LR *measurement with *f *= *PG *and *LR *= *μt *+ *l*, which is a modified form of Eq. (1). The following substitutions are also used

*G *= [(*μ*_*g *_+ *μ*_*f*_)*T *+ *l*_*g *_+ *l*_*f*_]

and

*H *= [(*μ*_*g *_- *μ*_*f*_)*T *+ (*l*_*g *_- *l*_*f*_)].

Taking partial derivatives of *f *with respect to each of the four parameters and *z *gives

$$\begin{array}{l} \frac{\partial f}{\partial \mu_{g}}=-100\frac{Tz}{H^{2}}-\frac{50T}{H}+50\frac{TG}{H^{2}},\\ \frac{\partial f}{\partial \mu_{f}}=100\frac{Tz}{H^{2}}-\frac{50T}{H}-50\frac{TG}{H^{2}},\\ \frac{\partial f}{\partial l_{g}}=-100\frac{z}{H^{2}}-\frac{50}{H}-50\frac{G}{H^{2}},\\ \frac{\partial f}{\partial l_{f}}=100\frac{z}{H^{2}}-\frac{50}{H}-50\frac{G}{H^{2}}, \end{array}$$

and

$$\frac{\partial f}{\partial z}=M.$$

These five partial derivative expressions were used to evaluate Eqs. (7–8).

### A. 2 Calibration map analysis

Numerical methods, idealizations, abbreviations, and quantities used previously [8] will be used to show the map is approximately linear. The normalized energy photon distribution is given by *p*(*ε*). The response for composite structure of *T *= total height corresponding to *t*_1 _cm of glandular tissue with Δ*t *= *T*-*t*_1 _cm of fatty tissue is given by

*z' *= ∫*p*(*ε*)exp[-*μ*_*g*_(*ε*)*t*_1 _- *μ*_*f*_(*ε*)Δ*t*]*dε*.

Attenuation coefficients are implicit functions of the x-ray energy (see previous work for details). The known *PG *= 100 × *t*_1_/*T*. Performing numerical integration using the 26 kVp – Mo/Mo simulated spectra for *t*_1 _= [1,2,3,4,5,6] cm with the known *PG *= [16.6, 33.3, 50.0, 66.6, 83.3, 100.0], gives R^2 ^= 0.999 when applying regression analysis to the ordered pairs: [ln(*z'*), *PG*]. The fitted line is shown in Fig. 5 for this example (regression line parameters as irrelevant here). Linear agreement is important because it validates the map form. The same analysis was carried out for *T *= [2,3,6] cm for all filter/target combinations and kVp values used for the phantom imaging based *LR *analysis with same the *t*_1 _values and *PG *sample points used to generate Fig. 6. For all combinations, the results were similar with R^2 ^= 0.999, which validates the use of Eq. (5).
